# Anticipating rotavirus vaccines – a pre-vaccine assessment of incidence and economic burden of rotavirus hospitalizations among children < 5 year of age in Libya, 2012-13

**DOI:** 10.1186/s12889-015-1400-7

**Published:** 2015-01-24

**Authors:** Salem Alkoshi, Eyal Leshem, Umesh D Parashar, Maznah Dahlui

**Affiliations:** Department of Social and Preventive Medicine, Faculty of Medicine, University of Malaya, Kuala Lumpur, Malaysia; National Center for Immunization and Respiratory Diseases, Centers for Disease Control and Prevention, Atlanta, Georgia USA; 17-4 pangasapuri permai putera, Jalan 13D, Desa Permai, Taman Dato, Ahmed Razali, 68000 Ampang Selangor, Malaysia

**Keywords:** Diarrhea, Rotavirus, Hospitalization, Incidence rate, Treatment cost, Libya

## Abstract

**Background:**

Libya introduced rotavirus vaccine in October 2013. We examined pre-vaccine incidence of rotavirus hospitalizations and associated economic burden among children < 5 years in Libya to provide baseline data for future vaccine impact evaluations.

**Methods:**

Prospective, hospital-based active surveillance for rotavirus was conducted at three public hospitals in two cities during August 2012 - April 2013. Clinical, demographic and estimated cost data were collected from children <5 hospitalized for diarrhea; stool specimens were tested for rotavirus with a commercial enzyme immunoassay. Annual rotavirus hospitalization incidence rate estimates included a conservative estimate based on the number of cases recorded during the nine months and an extrapolation to estimate 12 months incidence rate. National rotavirus disease and economic burden were estimated by extrapolating incidence and cost data to the national population of children aged <5 years.

**Results:**

A total of 410 children <5 years of age with diarrhea were enrolled, of whom 239 (58%) tested positive rotavirus, yielding an incidence range of 418-557 rotavirus hospitalizations per 100,000 children <5 years of age. Most (86%) rotavirus cases were below two years of age with a distinct seasonal peak in winter (December-March) months. The total cost of treatment for each rotavirus patient was estimated at US$ 679 (range: 200–5,423). By extrapolation, we estimated 2,948 rotavirus hospitalizations occur each year in Libyan children <5 years of age, incurring total costs of US$ 2,001,662 (range: 1,931,726-2,094,005).

**Conclusions:**

Rotavirus incurs substantial morbidity and economic burden in Libya, highlighting the potential value of vaccination of Libyan children against rotavirus.

## Background

Rotavirus is a major cause of severe diarrhea and hospitalization among children aged < 5 years worldwide. In 2008, globally rotavirus caused an estimated 453,000 deaths among children in this age group [1]; more than half of these deaths occurred in sub-Saharan Africa. In Libya, health service facilities in Libya are publicly owned and managed by the Ministry of Health. Public hospitals provide preventive and curative treatment to all citizens free of charge. While the Libyan government encourages the expansion of private health, private facilities have insufficient infrastructure to provide a full treatment service to severe diarrhea patients such as admission for intravenous fluid (IVF). Rotavirus disease accounted for 24 – 45% of diarrheal hospitalizations among children <5 during the period 1980-2009 [2,3]. The economic burden of rotavirus infections includes hospital costs, as well as indirect costs incurred by the society [4,5].

The World Health Organization (WHO) Strategic Advisory Committee on Immunization (SAGE) recommended adding rotavirus vaccine to all national immunization programmes, especially where the mortality rate of diarrhea affected up approximately 10% among children aged below 5 years [6-9]. Two live attenuated vaccines have been approved for global use: RotaTeq (RV5, Merck, Whitehouse Station, NJ, USA) is a pentavalent (G1, G2, G3, G4, P[8]) human-bovine reassortant vaccine and Rotarix (RV1, GlaxoSmithKline Biologicals, Rixensart, Belgium) is a monovalent (G1P[8]) vaccine derived from an attenuated human strain [10,11]. RotaTeq is administered at 2nd, 4th and 6th months of age, while Rotarix is administered at 2nd and 4th months of age. In 2009, the World Health Organization recommended the inclusion of rotavirus vaccine in the national immunization programs of all countries globally and particularly in those countries with high child mortality due to diarrhea [12]. In Libya, a live attenuated pentavalent vaccine based on a human rotavirus strain (RV5; RotaTeq™, Merck & Co. Inc., West Point, PA, USA) was introduced during October 1, 2013.

In February 2011, ongoing rotavirus surveillance activities in Libya were interrupted due to civil unrest. Our objective was to re-establish rotavirus surveillance to provide up-to-date estimates of the baseline pre-vaccine incidence of rotavirus hospitalizations among children aged < 5 years, and economic burden, in order to allow vaccine impact evaluations in the future.

## Methods

### Study design and setting

We conducted prospective, active, hospital-based surveillance for rotavirus-associated diarrheal hospitalizations among children < 5 years of age at three hospitals in two cities in Northwest Libya during the 9-month period from August 2012 to April 2013. These 3 hospitals are the only hospitals for treatment of severe diarrhea patients in the two cities, Khoms (estimated population 235,894) and Zliten (estimated population 239,860), which include a combined catchment population of 57,180 children aged < 5 years [13].

### Surveillance and data collection

Children <5 years of age with diarrhea symptoms (three or more instances of liquid stool in a day) who sought therapy in the pediatric ward at the study hospitals were identified and parental/guardian consent was obtained. Trained nurses collected stool samples from the suspected patients, whereas the staff researcher collected the demographic, clinical and economic data from patient’s files. The stool samples were transferred to the national laboratory at the National Center for Diseases Control (NCDC) where an enzyme immunoassay (ProSpect Rotavirus Test, Oxoid Ltd, UK) was used to detect Group A rotavirus.

Treatment cost of hospitalized rotavirus patients was calculated from perspectives of hospital (direct cost) and patient (indirect cost). Hospital cost was conducted only in Zliten hospital because all studied hospitals are reimbursed by the same source, Ministry of health and covered closely similar population. Hospital cost included three components: bed-day (Per Diem), medication and laboratory investigation tests. The cost of bed-day in the hospital includes the cost of staff salaries and the hotel cost, consisting of furniture, foods, laundry, disposal, cleaning, operation and maintenance. The cost of bed-day was calculated by dividing annual expenditure at the pediatric ward (US$ 983,015) by the number of patient’s days in the pediatric ward (accounted at 8,470 patient days in 2012). The cost of bed-day in the pediatric ward was provided from the financial management at the Zliten hospital during 2012. Medication cost was obtained from the central pharmacy, which was calculated separately for each patient, and the cost of laboratory tests were provided by the main laboratory in the hospital.

Several cost elements from the patient perspective were obtained from parents, including 1) the transportation cost for trips to the hospital when bringing or visiting the admitted patient; 2) household cost of expenditures related to the treatment of hospitalized rotavirus patients such as hygiene items for baby such as diapers; and 3) lost income of caregivers during the patient’s illness.

### Statistical analysis

We compared demographic and clinical characteristics of children hospitalized due to rotavirus diarrhea (stool tested positive for rotavirus) and diarrhea-hospitalizations not associated with rotavirus (tested negative for rotavirus). The annual incidence of rotavirus hospitalizations was calculated by dividing the number of rotavirus diarrhea hospitalizations by the number of children < 5 years of age residing in the catchment area of studies hospitals. We provided a conservative estimate calculated using the number of cases during the 9 months of enrolment as numerator (not inflating the number of rotavirus hospitalizations) to calculate annual incidence. Importantly, the historical months of the peak rotavirus season in Libya were captured during the 9 months of enrollment. Lastly, to estimate the annual number of rotavirus hospitalization countrywide and their associated costs, the conservative incidence and costs of rotavirus hospitalization from this study was extrapolated to the national population of 705,190 children <5 years of age in 2012 in Libya.

Data was analyzed by SPSS version 16. Chi-Square and P values <0.05 were considered statistically significant. Statistical tests were Chi-Square, X^2^ and t-test to obtain the outcomes such as mean, range and standard deviation. ANOVA or Mann–Whitney’s test were used to make a comparison between positive and negative-rotavirus cases. Mean, range and standard deviation were identified in economic data.

### Ethics

University of Malaya Medical Ethics Committee (IRP - 908.6), NCDC in Libya, and study hospitals provided ethical clearance to conduct the study.

## Results

**Figure 1:**
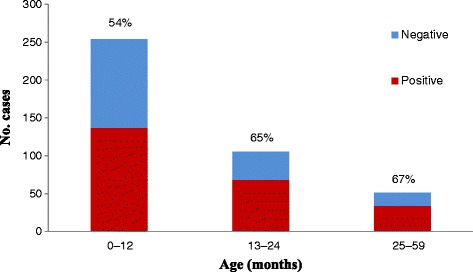
**Age distribution of positive and negative rotavirus cases association with hospitalization.**

**Figure 2:**
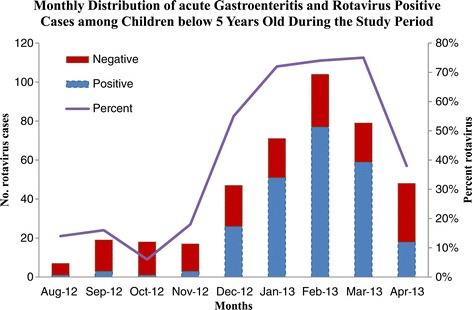
**Monthly distribution of positive and negative rotavirus cases association with hospitalization.**

A total of 410 children hospitalized due to diarrhea were enrolled, of whom 239 (58%) tested positive for rotavirus. Based on the catchment population in the studied hospitals (57,180 children aged < 5 years) in 2012, the unadjusted (conservative) incidence rate of rotavirus associated with hospitalization in the studied hospitals during the study period (9 months) was 418 per 100,000 (95% confidence interval, 405-431 per 100,000, Table 1). Applying the conservative estimate for incidence rate to whole population of Libya (705,190 children aged <5 years) was yielded an estimate of 2,948 (95% confidence interval, 2,845-3,048) national hospitalized rotavirus patients associated with hospitalization in 2012. Most (86%) patients with rotavirus diarrhea patients were under two years of age (Figure 1) and the disease showed a distinct winter seasonal peak during the months from December to March (Figure 2). Compared with rotavirus-negative patients, those who were rotavirus-positive were significantly more likely to suffer severe dehydration and vomiting (Table 2). Median duration of hospitalization of rotavirus patients was 3 days (range 1-15) as compared with 2 days (range 1-13) among rotavirus negative patients (p = 0.05), and nearly all patients receive intravenous fluids. No deaths were reported.

**Table 1:** **Conservative estimated number of rotavirus hospitalizations**

**Place of study**	**Population <5 years**	**Estimated number of rotavirus hospitalizations (95% confidence interval)**	**Annual estimated incidence/100,000 of rotavirus hospitalizations (95% confidence interval)**
		**Conservative estimate**	**Conservative estimate**
**Zliten and Khoms**	57,180	239	418 (405-431)
**Libya**	705,190	2,948 (2,845-3,084)	418 (405-431)

**Table 2:** **Basic characteristics of positive and negative rotavirus diarrhea associated with hospitalization below 5 years of age**

**Characteristics**		**Positive-rotavirus**	**Negative-rotavirus**	**p-value**
		**No. (%)**	**No. (%)**	
Rotavirus Cases		239 (58)	171 (42)	0.001
Places of Study (Districts)				
	Zliten	118 (49)	71 (42)	0.116
	Khoms	121 (51)	100 (58)	
Gender				
	Male	140 (59)	99 (58)	0.921
	Female	101 (41)	70 (42)	
Place of Living				
	Urban	62 (26)	42 (25)	0.752
	Rural	177 (74)	129 (75)	
Duration of Symptoms				
	<2 Weeks	233 (97)	165 (96)	0.810
	2 weeks and Above	6 (3)	6 (4)	
Length of Hospital Stay				0.000
	<7 Days	227 (95)	237 (99)	
	8 Days and Above	12 (5)	2 (1)	
Fever		154 (64)	118 (69)	0.335
Vomiting		233 (97)	146 (85)	0.000
Dehydration Degree				0.022
	Severe Dehydration	97 (41)	57 (33)	
	Moderate Dehydration	138 (58)	102 (60)	
	No Dehydration	4 (2)	12 (7)	
Rehydration Treatment				
	Treatment with IVF	237 (99)	168 (98)	0.404
	Treatment with ORT	2 (1)	3 (2)	

### Treatment costs

From the hospital perspective, the total expenditure to treat each rotavirus patient in the hospital was calculated to be US$ 488 (Intra quartile range [IQR], 318-541), the mean cost for each bed-day (Per Diem) was US$ 116 (Table 3), and the mean cost of medication was US$ 108 (IQR, 43-94). The most expensive medications were intravenous fluids and antibiotics which were given to 99% and 56% of rotavirus patients, respectively.

**Table 3:** **Variables used to calculate the per diem (Bed-Day) cost for hospitalized patients at Zliten Public Hospital, in 2012**

**Variable**		**Baseline**	**Source**
Per Diem Cost (Bed-Day)			
	Staff Salaries (US$)	752,266 (77%)	Zliten Hospital Management
	Hotel Cost (US$)	230,750 (23%)	Zliten Hospital Management
Total Per Diem Cost (US$)		983,015	
Average Length of Hospital Stay		3.02 days	This Study
Total Bed-Days at Pediatric Ward		8,470 days	Zliten Hospital Management
Costs per Day for each Patient (US$)		116	Zliten Hospital Management

Hotel Cost includes: Furniture, Equipment, Foods, Laundry, Disposal, Cleaning, Operation and Maintenance Costs.

From the family perspective, the mean cost for each hospitalized patient was approximately US$ 191 (IQR, 74-220). Family costs combined with hospital costs yielded an overall cost to treat each rotavirus patient of US$ 679 (IQR, 476-737).

Overall, hospital costs comprised 72% of the total expenditure, and family cost made up 28%. Hospital cost included bed-day (51%), medication (16%) and laboratory investigations (5%). Considering the family cost, transportation, household costs, and lost incomes were 12%, 10% and 6% of total cost, respectively (Table 4).

**Table 4:** **Treatment cost for hospitalized children associated with rotavirus diarrhea per each patient**

**Variable**		**Mean cost (US$)**	**%**	**SD (US$)**	**Range (US$)**
Hospital perspective					
	Per diem	349.31	51%	202.53	57–1,736
	Medication	107.73	16%	214.76	14–2,787
	Laboratory Tests	31.16	5%	10.62	14–92
Total		488.12	72%	427.91	85–4,615
Patient perspective					
	Transportation	78.42	12%	69.61	8–773
	Household	70.09	10%	69.46	16–472
	Lost income	42.37	6%	143.69	0–1,223
Total		190.88	28%	282.77	25–2,470
Overall Total		678.99		499.12	200–5,423

### National burden and costs of rotavirus hospitalization in Libya

Applying the conservative incidence rate for one year of surveillance yielded an estimate of 2,948 rotavirus hospitalizations annually in Libyan children <5 years of age. Combining these burden figures with cost data yielded a national economic burden of US$ 2,001,692 (range: 1,931,726-2,094,005).

## Discussion

Our prospective, active, hospital-based surveillance for rotavirus shows that, prior to rotavirus vaccine introduction, 58% of diarrheal hospitalizations among children < 5 years of age in Northwest Libya were caused by rotavirus. Each rotavirus hospitalization incurred a total cost of US$ 679, of which 72% were costs of hospital expenditures. By extrapolation, we estimated that nearly 3000 hospitalizations for rotavirus diarrhea occur each year in Libyan children 5 years of age or 1 in 50 children born each year is hospitalized for rotavirus by age 5, incurring total costs of US$ 2,001,662. This tremendous morbidity and economic burden highlights the potential value of vaccination of Libyan children against rotavirus.

Reports from Libya during the period 1980-2009 showed a rotavirus detection rate of 24% to 45% among children <5 years of age hospitalized with diarrhea [2,3,14-19], which were slightly lower compared with our data. This difference may have resulted from not including 3 months in our study during which rotavirus rates are usually lower, thereby increasing the proportion of rotavirus cases among the studied population; however, the detection rate of rotavirus in our study is also similar to other regional countries such as Oman (70%) and Iran (58%) [2]. Characteristics of rotavirus infection such as vulnerable ages to the disease, season of increased infection, difference in gender (male and female) and places of residence (urban and rural areas) were also comparable to previous reports of rotavirus disease in Libya [3,14,17-19].

Previous studies have not assessed the economic burden of rotavirus hospitalizations in Libya. Hospital costs in this study represented 72% of total costs of disease, which was higher than that reported in the U.S (66%) [20], but lower than in Brazil (86%) [21]. Lost income of caretaker in Hong Kong represented 10% of monthly income [22], while it was about 7% in Libya. These data illustrate the substantial economic burden of rotavirus on both the health care system and families.

Our study has some limitations. Notably, we had planned to continue surveillance for collection of data for at least one full calendar year; however, due to the political and security situation after the Libyan revolution, the study was interrupted in April 2013. Since the 3 months of data that we were not able to capture were late spring/summer months with lower rotavirus prevalence in previous years [23]. Also, since we conducted surveillance in only 2 cities in Libya, our findings may not be representative of the entire population. Nevertheless, these are among two of the large cities in Libya and their population structure and composition is similar to the rest of the country.

## Conclusion

We documented pre-vaccine incidence of rotavirus hospitalization in Libyan children and their associated costs to provide baseline data for future vaccine impact evaluations. Further studies of rotavirus disease burden among hospitalized children, in the form of active hospital based surveillance in Libya, will be crucial to understand the effect of introducing the vaccine into national childhood immunization, to provide scientific evidence for continued immunization efforts and identification of barriers for vaccine impact and effectiveness [24,25].
